# PROTOCOL: Attitudinal Factors Related to the Use of Digital Technologies in Health by Older Adults: An Overview of Reviews

**DOI:** 10.1002/cl2.70022

**Published:** 2025-02-26

**Authors:** Elzbieta Bobrowicz‐Campos, Cristina Camilo, Guilherme Galhardo Pinheiro

**Affiliations:** ^1^ Centre for Psychological Research and Social Intervention, Iscte – University Institute of Lisbon Lisbon Portugal

**Keywords:** attitudes and behaviours, digital divide, digital health technologies, digital transformation, older adults, overview of reviews

## Abstract

This is the protocol for a Campbell systematic review. The objective is as follows: to consolidate the available evidence on attitudinal aspects related to the utilisation of digital technologies in health among older adults. More specifically, we will summarise and systematise the existing reviews findings to identify attitudinal factors that interfere with the use of digital technologies in health in advanced age and to determine whether these factors act as facilitators or barriers. We will also compare the influence of attitudinal factors on technology use behaviour, considering the type of technology in question, and the purpose and context of its use. The overview of reviews questions are the following: (1) What are the attitudinal factors related to the use of digital technologies in health by older adults? (2) Which of these factors facilitate the use of digital technologies in health, and which make it difficult? (3) Are the attitudinal factors that facilitate and make difficult the use of digital technologies in health different for different types of technologies? (4) Are the attitudinal factors that facilitate and make difficult the use of digital technologies in health different for different purposes and contexts of use of these technologies?

## 1 Background

In recent years, there has been exponential growth in the use of digital technologies for the most diverse purposes and an accelerated transfer of services (e.g., services provided by public administration, banking or commercial services, services of leisure and entertainment) and networks (either formal and informal, including professional networks or creative and community‐based networks that bring together people with common interests or hobbies) to the digital space, with both having an increasing impact on decision‐making and successful performance of activities in different areas of life. The changes driven by digital transformation can be observed at social, economic, and cultural levels, through the successive increases in the rates of use of digital equipment and connectivity, daily time spent with media, quantity and variety of devices owned, and money spent on digital media downloads and subscriptions or online purchases, among others (DataReportal, We Are Social, Hootsuite, and Kepios 2022). It is expected that digital transformation will bring multiple benefits to people, enriching their lives, offering new resources and opportunities for their development, and improving their well‐being (European Commission 2020; Organization for Economic Co‐operation and Development 2019). However, these benefits may not reach everyone equally, as there are still a significant number of people who cannot keep up with digital and technological progress. For example, in 2022, Internet adoption worldwide varied from 24% in Central Africa to 98% in Northern Europe, with almost three billion people remaining offline (DataReportal, We Are Social, Hootsuite, and Kepios 2022). The lowest percentages of internet usage were registered in low‐income countries (27%), as well as in least developed countries and landlocked developing countries (35% and 39%, respectively) (International Telecommunication Union 2023). Low adoption of digital and technological innovation may lead to digital exclusion, compromising participation in the economic, social, and political life of society and, consequently, contributing to replicating or exacerbating existing offline inequalities (Ragnedda et al. 2022; van Deursen et al. 2021).

### 1.1 Factors Related to the Adoption of Digital and Technological Innovation

There are several reasons for the low adoption of digital and technological innovation, the most important of which are limited availability, accessibility and/or affordability of digital connectivity and infrastructure, limited digital literacy competences, and/or limited digital engagement (Olphert and Damodaran 2013; van Deursen et al. 2017; van Deursen and van Dijk 2015). The barriers to accessing and using digital technologies limit the opportunities to benefit from them for personal and professional development, thereby leading to a digital divide. The phenomenon of the digital divide can be analysed at multiple levels, depending on the main reason behind it. Thus, the access divide is described as the first‐level digital divide, the digital literacy divide as the second‐level digital divide, and the divide in goal‐oriented and engaged use of digital technologies as the third‐ and fourth‐level digital divide (Olphert and Damodaran 2013; Scheerder at al. 2017). The digital divide seems to depend on the influence of different sociodemographic, economic, material, socio‐cultural, and person‐related factors (Scheerder et al. 2017). Among the factors mentioned, age has been consistently indicated as a key characteristic in the analysis of digital exclusion, with older adults (compared to young and middle‐aged adults) being the ones who show the greatest gap in reaching benefits resulting from digital transformation (European Commission 2022; International Telecommunication Union 2023). The COVID‐19 pandemic has highlighted the importance of this age‐related gap and revealed the harmful effects it can have (Seifert et al. 2021).

Currently, there are many initiatives aimed at digital empowerment of older adults. However, despite substantial efforts undertaken to create opportunities for improving digital literacy competences in the age group in question, the participation of older adults in digital education programmes is still insufficient (UNESCO Institute for Lifelong Learning 2019). There are also significant gaps in the generalised, proactive, and transformative uptake of the learning effects in everyday activities (Arthanat et al. 2019; Schreurs et al. 2017; Steelman et al. 2016). The success of digital education programmes and successive engagement with digital technologies seem to depend, among others, on individual motivation (e.g., attitudes towards digital media) personal characteristics (e.g., curiosity, confidence, and self‐efficacy), and needs that guide activities carried out through digital technologies (e.g., leisure and objective‐oriented activities) (Arthanat et al. 2019; Rivinen 2020; Tirado‐Morueta et al. 2020). Previous experience in using digital technologies (in terms of quantity and quality) for obtaining information, communicating, and interacting with others, carrying out transactions, dealing with administrative issues, and consuming leisure and entertainment, is also recognised as relevant to digital (dis)engagement (Arthanat et al. 2019; Llorente‐Barroso et al. 2015; Schreurs et al. 2017; Steelman et al. 2016). According to Nadal et al. (2020), the actual use of digital technology and the user engagement are essential to moving from the initial stages of the technology acceptance lifecycle (that are pre‐use acceptability and initial use acceptance) to the final stage. which refers to the adaptation and sustained use acceptance.

### 1.2 Digital Transformation in Healthcare

Digital transformation gains a special relevance in the context of health. It is considered a key factor in improving healthcare quality, by enabling the redefinition of the care model to be more integrated, more participatory, and more personalised (European Commission 2012, 2018). The anticipated benefits encompass structures (in terms of improving their accessibility, inclusiveness, efficiency, and sustainability), processes (in terms of streamlining health and disease management according to individual preferences, needs and values, and considering contextual specificity), and outcomes (which is due to the existence of integrated and proximity care) (World Health Organization 2021). It is for these reasons that, in recent years, the organisational and cultural change that involves the integration of digital technologies in healthcare (in short: digitisation of healthcare; Iyamu et al. 2021) has become more widespread (European Commission 2022). At the same time, an exponential increase in the value of the digital health market and the number of digital health users has been registered (DataReportal, We Are Social, Hootsuite, and Kepios 2022). The digitalisation of healthcare can be observed through the increasing delivery of health‐related information, products, and services with the support of different types of technologies (e.g., mobile or nonmobile, with or without Internet access) and devices (e.g., computers, smartphones and tablets, wearables) (de Santis et al. 2023). It is also manifested through the increasing technology‐mediated interaction between healthcare providers and beneficiaries, which includes the use of social media, mobile applications, and websites for informing, screening, diagnosis, treatment, and monitoring purposes (de Santis et al. 2023; Castiglia et al. 2023). Adopting digital technologies in healthcare is expected to improve the efficiency and sustainability of health systems (Bobrowicz‐Campos and Matos 2020).

However, digital transformation in healthcare can also contribute to the worsening of social inequalities, if the principles of inclusivity and social responsibility are not respected, or if the different expectations and capabilities of citizens are not accommodated, leading to increased unmet health needs, and compromising well‐being and quality of life (Robinson et al. 2015). This risk is particularly significant for older adults who, due to age‐related changes in multiple physiological systems (Clegg et al. 2013), have an increased need to resort to health services and, at the same time, show greater difficulties in adopting digital and technological innovation (European Union 2020). To illustrate, estimates for 2017 and 2018 show that among European Union citizens aged 65–74 years, more than 50% were affected by a long‐standing illness or health problem, and within a year before data collection, more than 85% had consulted a general medical practitioner, and nearly 70% had consulted a surgical practitioner (European Union 2020). This share was significantly higher for people aged 75 years or more. During the same period, 44% of European Union citizens aged 65–74 claimed never using a computer, and only 44% in this age group had recent experience of sending and receiving e‐mails, 24% of using a telephone or video calls over the internet to communicate, and 18% of making use of social networks (European Union 2020). This share was significantly lower for people aged 75 years or more. Even though these indices are improving year after year, there is still a large number of older adults who are not able to use digital technologies autonomously or proactively. For digitally excluded older adults, the large‐scale replacement of in‐person services with digital services, accompanied by the diffusion of patient‐oriented and technology‐based digital solutions that aim to encourage individual participation in health‐related decision‐making, may increase barriers to accessing healthcare and reduce this healthcare quality (Bobrowicz‐Campos and Matos 2020). That is why it is so important to understand the attitudinal factors that interfere with the use of digital technologies in health by older adults. Deepening knowledge of this topic can help define solutions that promote behavioural change with respect to the adoption of digital technologies for health‐related purposes in the age group in question.

Individual attitudes are recognised as a potential factor that can impede or hinder the integration of digital health technologies (Arning and Ziefle 2009). This recognition stems from the conceptualisation of attitudes. The theory of planned behaviour, proposed by Ajzen (1985, 1991), posits that behaviours are influenced by immediate determinants such as behavioural intentions and, under specific circumstances, perceived behavioural control. Behavioural intentions, in turn, are shaped by a combination of three factors: attitudes toward the behaviour, subjective norms, and perceived behavioural control. Individuals' attitudes are formed based on their feelings and evaluations of a particular behaviour. For instance, whether they perceive the outcomes of engaging in digital health actions as positive or negative will impact their intention to perform such behaviours.

Attitudes are relatively stable evaluations, ranging from negative to positive, and are influenced by specific beliefs, emotions, and past behaviours associated with the object of evaluation. An illustrative example of their significant impact on behaviour comes from a study on telemedicine, which found that 42% of participants who had never used telemedicine preferred in‐person doctor visits (Beck 2016, June 26). These findings underscore how attitudes provide crucial insights into behaviour, serving as a substantial barrier to the widespread adoption of health technology.

Several reviews have summarised evidence related to the use of digital technology by older adults for health‐related purposes. However, in most cases, these reviews were confined to one specific technology (e.g., Ahmad et al. 2022) or one specific context (e.g., Fjellså et al. 2022), or they were focused on a certain population (Walker et al. 2017) or a limited set of factors (Hasnan et al. 2022). Although the existing reviews have provided relevant insights into the phenomenon of interest, their focused interest does not allow obtaining an exhaustive view of the person‐related factors that interfere with the use of digital technologies in health by older adults, nor do they allow understanding whether these factors act consistently as facilitators or hinderers, or whether they change depending on the type of technology, purpose of its use or context. This overview of reviews intends to fill this gap and address the phenomenon under study from a broader perspective by aggregating and systematising findings from multiple reviews and by providing a comprehensive appraisal of these findings. This will certainly strengthen the understanding of the topic, opening new horizons for research on determinants of the multiple‐level digital divide, which in turn will contribute to the elaboration of recommendations to inform educational and health and social care‐related practices and support decision‐making aimed at digital equity in health.

A preliminary search of the JBI Evidence Synthesis database, the Cochrane Database of Systematic Reviews, and the Centre for Reviews and Dissemination Database has revealed that there is currently no overview of reviews (neither published nor in progress) examining the relationship between attitudinal factors and intention to use digital technologies in health in advanced age.

## 2 Objectives

The main objective of this overview of reviews is to consolidate the available evidence on attitudinal aspects related to the utilisation of digital technologies in health among older adults. More specifically, we will summarise and systematise the existing reviews findings to identify attitudinal factors that interfere with the use of digital technologies in health in advanced age and to determine whether these factors act as facilitators or barriers. We will also compare the influence of attitudinal factors on technology use behaviour, considering the type of technology in question, and the purpose and context of its use.

The overview of reviews questions are the following:

1. What are the attitudinal factors related to the use of digital technologies in health by older adults?
2. Which of these factors facilitate the use of digital technologies in health, and which make it difficult?
3. Are the attitudinal factors that facilitate and make difficult the use of digital technologies in health different for different types of technologies?
4. Are the attitudinal factors that facilitate and make difficult the use of digital technologies in health different for different purposes and contexts of use of these technologies?

## 3 Methods

This overview of reviews will follow the JBI methodology for umbrella reviews of quantitative and qualitative evidence (Aromataris et al. 2015, 2020). To ensure a transparent, complete, and accurate account of the review process, the Preferred Reporting Items for Overviews of Reviews (PRIOR) guidelines (Gates et al. 2022) and an updated Preferred Reporting Items for Systematic Reviews and Meta‐Analyses (PRISMA) extension for review protocol checklist (Shamseer et al. 2015) will be used (Table 1).

**Table 1:** An updated Preferred Reporting Items for Systematic Reviews and Meta‐Analyses (PRISMA) extension for review protocol checklist (Shamseer et al. 2015).

Section and topic	Item no	Checklist item	Reported on page #
**ADMINISTRATIVE INFORMATION**			
Title:			
Identification	1a	Identify the report as a protocol of a systematic review	1
Update	1b	If the protocol is for an update of a previous systematic review, identify as such	N/A
Registration	2	If registered, provide the name of the registry (such as PROSPERO) and registration number	10
Authors:			
Contact	3a	Provide name, institutional affiliation, e‐mail address of all protocol authors; provide physical mailing address of corresponding author	1
Contributions	3b	Describe contributions of protocol authors and identify the guarantor of the review	23
Amendments	4	If the protocol represents an amendment of a previously completed or published protocol, identify as such and list changes; otherwise, state plan for documenting important protocol amendments	N/A
Support:			
Sources	5a	Indicate sources of financial or other support for the review	22–23
Sponsor	5b	Provide name for the review funder and/or sponsor	N/A
Role of sponsor or funder	5c	Describe roles of funder(s), sponsor(s), and/or institution(s), if any, in developing the protocol	N/A
**INTRODUCTION**			
Rationale	6	Describe the rationale for the review in the context of what is already known	2–9
Objectives	7	Provide an explicit statement of the question(s) the review will address with reference to participants, interventions, comparators, and outcomes (PICO)	9
**METHODS**			
Eligibility criteria	8	Specify the study characteristics (such as PICO, study design, setting, time frame) and report characteristics (such as years considered, language, publication status) to be used as criteria for eligibility for the review	10–13
Information sources	9	Describe all intended information sources (such as electronic databases, contact with study authors, trial registers or other grey literature sources) with planned dates of coverage	12–16
Search strategy	10	Present draft of search strategy to be used for at least one electronic database, including planned limits, such that it could be repeated	13–14 Appendix SI
Study records:			
Data management	11a	Describe the mechanism(s) that will be used to manage records and data throughout the review	16
Selection process	11b	State the process that will be used for selecting studies (such as two independent reviewers) through each phase of the review (i.e., screening, eligibility and inclusion in meta‐analysis)	16–17
Data collection process	11c	Describe planned method of extracting data from reports (such as piloting forms, done independently, in duplicate), any processes for obtaining and confirming data from investigators	19–20
Data items	12	List and define all variables for which data will be sought (such as PICO items, funding sources), any pre‐planned data assumptions and simplifications	Appendix SII
Outcomes and prioritisation	13	List and define all outcomes for which data will be sought, including prioritisation of main and additional outcomes, with rationale	Appendix SII
Risk of bias in individual studies	14	Describe anticipated methods for assessing risk of bias of individual studies, including whether this will be done at the outcome or study level, or both; state how this information will be used in data synthesis	N/A
Data synthesis	15a	Describe criteria under which study data will be quantitatively synthesised	20–21
	15b	If data are appropriate for quantitative synthesis, describe planned summary measures, methods of handling data and methods of combining data from studies, including any planned exploration of consistency (such as *I* ^2^, Kendall's *τ*)	20–22
	15c	Describe any proposed additional analyses (such as sensitivity or subgroup analyses, meta‐regression)	N/A
	15d	If quantitative synthesis is not appropriate, describe the type of summary planned	20–22
Meta‐bias(es)	16	Specify any planned assessment of meta‐bias(es) (such as publication bias across studies, selective reporting within studies)	19–20
Confidence in cumulative evidence	17	Describe how the strength of the body of evidence will be assessed (such as GRADE)	N/A

The title of this overview of reviews was registered with Campbell with the identifier (ID): cl2.20240039.

### 3.1 Eligibility Criteria

#### 3.1.1 Participants

This review of reviews will include reviews that focus on males and females, aged 60 years or more, professionally active or not, with different economic statuses, residing in the community or institutions (e.g., long‐term care institutions) and benefitting or not from institutionalised care and support (e.g., adult day centres, social centres, home support services), residing in urban and rural areas of any region in the world, healthy or with different clinical conditions, as long as these conditions are not clinically diagnosed intellectual developmental disorders (i.e., different aetiological subtypes of intellectual disability, including Fragile X Syndrome, foetal alcohol spectrum disorder, Prader‐Willi Syndrome, among others) or major neurocognitive disorders (i.e., different aetiological subtypes of dementia, including Alzheimer's disease, vascular dementia, frontotemporal dementia, Lewy body disease, among others). Reviews targeting mixed populations (e.g., different age groups) will only be included if the review findings related to older adults can be extracted and if these data have not been captured in another included review.

#### 3.1.2 Concept

This overview of reviews will consider reviews that report on attitudes toward behaviour, beliefs about costs and benefits, subjective norms, and perceived behavioural control regarding the use of digital technologies in health.

Attitudes can be conceptualised as ‘a psychological tendency that is expressed by evaluating a particular entity with some degree of favour or disfavour’ (Eagly and Chaiken 1993, 1). Under particular circumstances individuals' attitudes guide their behaviour toward the attitude object, meaning that attitudes toward a particular digital technology in health guide the intention to use that specific technology. Therefore, reviews focusing on attitudinal factors that interfere with the use or intention to use digital technologies in health will be included in this overview of reviews. On the other hand, we will exclude reviews that describe only technology‐ (e.g., referring to the level of suitability of certain technology for persons with sensory and motor changes) or context‐related factors (e.g., concerning the level of availability of a certain technology in settings of interest) associated to digital health technology use or intention to use. We will also exclude reviews that report on person‐related factors other than attitudinal (e.g., alluding to education levels as responsible for use or non‐use of a certain technology).

In terms of digital technologies, we consider them to be the electronic tools, systems, devices, and resources that generate, store, or process data (Kebede et al. 2022), as well as artificial intelligence tools that can learn from experience and improve performance adapting to new information, without being explicitly programmed (Murphy et al. 2021). For this overview of reviews, we will consider reviews that focus on different digital technologies such as mobile and nonmobile technologies (with and without Internet access), different digital devices (e.g., computers, smartphones and tablets, wearables) and artificial intelligence technologies (e.g., machine learning or deep learning), among others, that are used to advance healthcare, prevent illnesses, or provide treatment (de Santis et al. 2023). This encompasses a diverse range of approaches, including screening and monitoring tools, as well as counselling through digital media. Digital solutions that support remote interaction between healthcare providers and users (e.g., social media, mobile applications, websites, emails, text messages used for virtual medical appointments, teleconsultations, telemonitoring, or prescription management) will also be considered (de Santis et al. 2023). On the other hand, we will exclude from this overview of reviews all reviews that focus on the use of digital technologies from a perspective that does not consider health‐related purposes.

#### 3.1.3 Context

This overview of reviews will include reviews that consider any setting, whether community (e.g., place of residence, place of work, community centre), health care (e.g., inpatient or outpatient care facilities), or social care (e.g., residential homes or day centres). There will also be no restrictions regarding geographical location or cultural, ethnic, or socioeconomic context.

#### 3.1.4 Types of Sources

In this review of reviews, systematic reviews, scoping reviews, meta‐analyses and narrative reviews of quantitative, qualitative, and mixed‐method studies will be considered if they provide clearly defined review question(s) and eligibility criteria to select primary studies and as long as they describe in detail the strategy of the search and selection process that includes at least one bibliographic database.

### 3.2 Search Strategy

The search strategy will aim to locate published and unpublished reviews of quantitative, qualitative, and mixed‐method studies, with or without meta‐analyses or meta‐synthesis. Following JBI recommendations for umbrella reviews of quantitative and qualitative evidence (Aromataris et al. 2015, 2020), we used a three‐step search strategy to define a draft set of search terms and search strings. Namely, in the first step, we performed an initial limited search of Academic Search Complete, APA PsycArticles, APA PsycInfo, E‐Journals, MEDLINE, and Psychology and Behavioural Sciences Collection (all via EBSCOhost) to identify relevant articles on the topic of interest of this overview of reviews. Then, the titles and abstracts of these articles, as well as the index terms used to describe them, were analysed (second step). This process allowed us to identify the text words and index terms that match the inclusion criteria for this overview of reviews. Based on the collected information, we developed a draft of the complete search strategy for the final databases (third step). The draft search strategy was agreed upon by the review team members. Then, it was analysed by an information specialist for validation and refinement purposes.

The search strategy incorporates search terms corresponding to inclusion criteria, organised into four themes: participants (e.g., older*, elder*, geriatric*, aging, ageing), concept of digital technology in health (e.g., ‘digital health’, ‘electronic health’, ‘mobile health’, ‘digital technolog*’, ‘mobile technolog*’, ‘smart technolog*’), concept of attitudinal factors (e.g. attitude*, belief*, norm*, opinion*, perception*, value*) and types of sources (review*, meta‐analys*, meta‐synthes*, ‘evidence‐based analys*’, ‘evidence‐based synthes*’). Since no setting‐related, geographical, cultural, ethnic, or socioeconomic restrictions were defined for the context, it was not considered when developing the search strategy.

To capture all available evidence, different terminologies and various spelling of search terms were considered. Regarding search strings, terms belonging to the same theme were combined with OR, and terms belonging to different themes were combined with AND. The search for terms will cover the title, abstract and subject heading fields. A detailed example of the search strategy can be found in Appendix SI. This search strategy will be customised for each included database and information source.

The search strategy will include a date and language restrictions. Namely, it will consider published and unpublished reviews written since 2005. The year 2005 was chosen for two reasons. First, it was the year in which the WHO adopted Resolution WHA58.28, recognising the potential of using information and communication technologies to improve quality, safety, and access to healthcare, and encouraging the integration of eHealth into healthcare systems and services (World Health Organization 2005). Second, it was the year in which the WHO launched the Global Observatory for eHealth to monitor the evolution and impact of the use of information and communication technologies for health and health‐related outcomes around the world (World Health Organization 2006). Both initiatives have significantly contributed to the increase in research into the technology‐driven transformation of healthcare and the opportunities and challenges associated with this transformation. Reviews dated before the selected date will be excluded.

About language restriction, only published and unpublished reviews written in English will be considered. While we acknowledge that this approach may limit access to potentially relevant reviews developed in cultural and socioeconomic contexts different from those covered here, potentially underrepresenting certain local and regional realities, we believe that the majority of English‐language reviews synthesise evidence from diverse contexts and realities, providing a sufficiently comprehensive perspective on the phenomenon under study. Our decision is also related to the fact that the inclusion of sources written in languages other than English raises problems of a practical and methodological nature, significantly reducing the feasibility of the review process (due to time and resource constraints), as well as its transparency (due to limited possibility of verification and replication of the search strategy). In this sense, we consider that reviews not written in English should be excluded from this overview of reviews; however, in the final report, we will discuss the implications of the decision made.

Aiming at providing complete and relevant multidisciplinary coverage, the final databases to be searched will be from the areas of medical and social sciences and will include PubMed, CINAHL Complete via EBSCOhost, Web of Science Core Collection, Scopus, APA PsycArticles via EBSCOhost, APA PsycInfo via EBSCOhost and AgeInfo through Centre for Policy on Ageing website. The review registers, such as JBI Evidence Synthesis, Cochrane Database of Systematic Reviews, University of York Centre for Reviews and Dissemination Database, and Epistemonikos, will also be searched.

To capture relevant grey literature, including standalone publications, organisation reports, and dissertations, we will consult the DART‐Europe E‐theses Portal, Global ETD Search, Open Access Theses and Dissertations (OATD), and EBSCO Open Dissertations.

Supplementary search methods will also be implemented to ensure comprehensive coverage. Namely, the reference lists of all included sources of evidence will be scrutinised for additional studies of potential interest. For forward citation tracking, we will use citation databases (such as Google Scholar) to perform forward citation tracking of all included reviews. This will help identify any relevant studies that have cited the included reviews since their publication. In addition, we will conduct targeted web searches, focusing on professional and academic websites that are relevant to the field, to uncover any additional reviews or grey literature not indexed in traditional databases. Finally, if necessary, we will reach out to key authors in the field to enquire about any ongoing or unpublished reviews that could contribute to our overview.

All stages of the search process will be duly described in the final report.

### 3.3 Study Selection

Following the search, all identified citations will be collated and uploaded into Rayyan, a research collaboration platform designed to support conducting of literature reviews in terms of records organisation and management (Ouzzani et al. 2016). Then, duplicates will be removed and a pilot screening of 20 randomly selected records will be conducted based on pre‐defined instructions for title and abstract screening, defined according to this review eligibility criteria. The results of the pilot screening will be analysed by the review team to clarify potential ambiguities, ensure relevance to the review objectives, and maintain consistency in applying criteria for inclusion and exclusion. If necessary, screening instructions will be refined. Once screening instructions are clarified and/or refined, the remaining titles and abstracts will be screened for compliance with the review inclusion criteria. This process will be assured by two independent reviewers. To discuss and address any challenges or uncertainties that may arise in the screening process, regular meetings of the review team will be held. After the screening process is complete, discordant decisions will be identified and resolved through discussion between the two reviewers or with the help of the third reviewer. However, if uncertainty or disagreement persists, the record will be included in the full‐text screening phase.

In the next step, potentially relevant records will be retrieved in full and assessed against the review inclusion criteria, using a detailed assessment guide. Also in this case, the process will begin with the pilot screening of a random sample of full‐text records, following procedures similar to those described for the pilot screening of titles and abstracts. The assessment process will be conducted by two independent reviewers. However, regular meetings of the review team will be held to ensure consistency in the process and address any challenges or uncertainties faced. Discrepant decisions will be analysed and resolved through discussion between review team members until a consensus is reached. Full‐text records excluded from the review for not meeting the eligibility criteria, as well as the reasons for their exclusion, will be documented in the final report of this overview of reviews.

**Figure 1:**
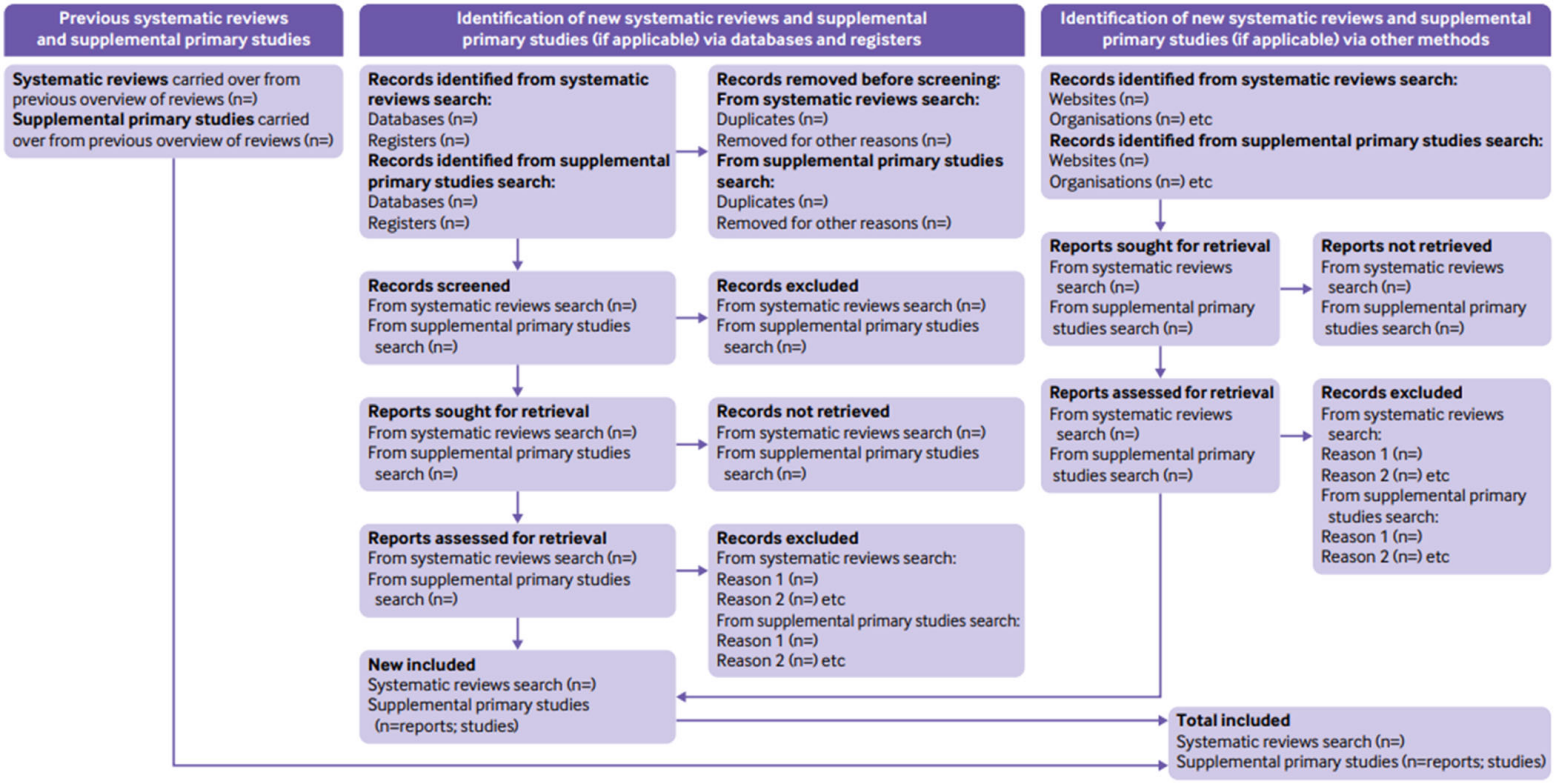
PRIOR flow diagram template for overviews of reviews (Gates et al. 2022).

The final report will present the results of the search and selection processes in full (Figure 1), through the narrative and the PRIOR flow diagram for overview of reviews (Gates et al. 2022).

### 3.4 Critical Appraisal of Methodological Quality

To assess the methodological quality of the included reviews and establish the extent to which each of these reviews had controlled the risk of bias in their design, conduct, analysis, and findings presentation, the retrieved records will be subjected to critical appraisal. Critical appraisal will be performed by two independent reviewers using the JBI's critical appraisal checklist for systematic reviews and research synthesis (Aromataris et al. 2020). The choice of this checklist is related to the fact that this overview of reviews is conducted under the JBI methodology for comprehensive reviews of quantitative and qualitative evidence. The critical appraisal checklist of interest consists of 11 items that question the adequacy of the review process and the clarity of its reporting. For each item there will be four response options, including (i) ‘yes’, assigned in a situation where the options taken by the authors of the review under analysis are appropriate; (ii) ‘no’, assigned in a situation where the options taken by the authors of the review under analysis are not appropriate or where there is no information that addresses the item in question; (iii) ‘unclear’, assigned in a situation where the data provided are not sufficient to give a ‘yes’ or ‘no’ answer; and (iv) ‘not applicable’, assigned in a situation where the given item can't be answered because the requested information does not apply to the review under analysis. Any disagreements that arise between the reviewers will be resolved through discussion. If necessary, assistance from a third reviewer will be requested. The JBI's critical appraisal results will then be classified into categories of high quality (for reviews with a sum of 9 to 11 ‘yes’ answers), moderate quality (for reviews with a sum of 6 to 8 ‘yes’ answers), low quality (for reviews with a sum of 3 to 5 ‘yes’ answers) and very low quality (for reviews with a sum of 0 to 2 ‘yes’ answers). The critical appraisal results will be presented in narrative and tabular format in the final report of the overview of reviews.

As this overview of reviews will consider different types of sources and with varying levels of methodological robustness (e.g., systematic reviews and scoping reviews), the results of critical appraisal will not be used for inclusion and exclusion purposes. This means that all reviews, regardless of their methodological quality, will be subject to the data extraction and data summary processes. However, the critical appraisal results will be considered to guide interpretations of the overview of reviews' findings and inform its strengths and weaknesses.

The results of critical appraisal of methodological quality will be presented in tabular format, accompanied by a narrative.

### 3.5 Data Extraction

Before starting the extraction process, the reviews included in this overview of reviews will be analysed for overlapping primary studies. To manage the existing overlap, a citation matrix will be developed, and the corrected covered area index will be calculated (Pieper et al. 2014). The frequency of overlapping studies will be noted in the final report of the overview of reviews.

Data from the included reviews will be extracted by two independent reviewers using a data extraction tool developed by the review team members, based on the JBI data extraction form for review for systematic reviews and research synthesis (Aromataris et al. 2020). Data extracted will include details describing each review (e.g., type of review, review objectives, number and publication date range of studies in the review, checklist used for critical appraisal) and referring to the population (e.g., sample size, age, gender, health condition), concept (e.g., attitudinal factors addressed by reviews, type of digital health technologies addressed by reviews, method of the analysis), context (e.g., countries, contexts and settings where the studies were conducted) and key findings relevant to the review question (Pollock et al. 2023). In cases where additional data is needed, the review authors will be contacted to provide all necessary information.

A draft data extraction tools can be found in Appendix SII. To enhance the effectiveness of the data extraction process and minimise the risk of errors, the extraction strategy will be developed collaboratively by all members of the review team and tested through a pilot extraction of five reviews using draft data extraction tools. The pilot testing outputs will be carefully analysed to determine whether the draft extraction tools are logically organised and whether the guidelines for their use are understandable to each reviewer. At the same time, it will be verified whether all relevant data to answering the research questions of this overview of reviews are properly identified and accurately charted. If necessary, the draft data extraction tools will be modified and revised. Modifications will be detailed in the final report of the overview of reviews for transparency.

Any disagreement between the reviewers regarding data extraction will be solved through discussion. If consensus cannot be reached, a third reviewer will be consulted.

### 3.6 Data Summary and Synthesis

Data obtained from the included reviews will be systematically organised into tables and supplemented by a narrative synthesis to address the review questions in line with the established inclusion criteria. All data will be subject to double data entry to ensure accuracy and reliability. Data from quantitative reviews and qualitative reviews will be presented separately, being organised based on the following classification categories: (i) attitudinal factors related to the user perceptions, beliefs, and experiences regarding digital technologies in health; (ii) digital health technologies classified into different categories of tools and interventions; and (iii) specific purposes and contexts in which these technologies are used. For quantitative data, existing associations between attitudinal factors and specific types of digital health technology will be additionally detailed. This will include reporting the significance, direction, and strengths of these associations, enhancing the interpretability of the findings. Given that the interest of this overview of reviews lies in aggregating and systematising findings from reviews that focus on the use of different digital health technologies, for different purposes and in different contexts, we believe that statistical pooling of quantitative data will not be possible.

After being summarised, the quantitative and qualitative data will be analysed, critically compared, and then discussed in terms of their convergence, complementarity, or divergence. This will involve assessing how different studies align or differ in their results and conclusions, providing deeper insight into the overall evidence base. To this end, the pilar integration process (Johnson et al. 2019) will be used, consisting of four sequential stages that include: (i) listing relevant quantitative and qualitative data; (ii) matching similar data from quantitative and qualitative data sets and organising them into categories; (iii) cross‐checking data for completeness and appropriateness of match and identifying emerging patterns; (iv) and pilar building through comparing and contrasting the findings from the previous stages and integrating them into a meaningful narrative. To support the credibility, confirmability, and transferability of this narrative, its construction will be framed within the theory of planned behaviour (Ajzen 1985, 1991) that will be used to explain the findings in a theoretically grounded manner, identifying patterns of attitudes, subjective norms, and perceived behavioural control that shape intentions and behaviours, and to situate these findings in the literature on the subject of interest (Bingham 2023). All stages of the pillar integration process will be carried out collaboratively by the entire review team.

This approach will provide a comprehensive understanding of the current evidence regarding digital health technologies and their associated attitudinal factors. More specifically, it will allow identifying how different attitudinal factors may manifest and influence user intention and behaviour across different digital health technologies and how specific features of digital technologies, alongside the broader attitudinal factors, impact user interactions and behaviours. By focusing on both general attitudinal factors and the unique aspects of different technologies, we will provide insights into the complex relationship between user attitudes and technology use behaviour.

Integrated and theoretically grounded findings of the overview of reviews, highlighting key themes, gaps in the literature, and areas of consensus and contention will be presented in a ‘Summary of Findings’ table. This table will also include the quality evaluation rates given based on methodological limitations of the included reviews, as well as their consistency, risk of bias, and relevance to the population of interest.

## Author Contributions

All authors developed the research questions, objectives of the study, and study design. E.B.‐C. and C.C. planned the methodology of the study. All authors developed the search strategy. E.B.‐C. performed the search. E.B.‐C. developed the data charting form. All authors approved the final text of the protocol sent to be published.

E.B.‐C. will be responsible for the final report of the review described in this protocol.

## Conflicts of Interest

The authors declare no conflicts of interest.

## Supporting information

Supporting information.
