# Supplementary material for: PROTOCOL: Attitudinal Factors Related to the Use of Digital Technologies in Health by Older Adults: An Overview of Reviews
Source: Campbell Syst Rev. 2025 Feb 26;21(1):e70022. doi: 10.1002/cl2.70022 (PMC11865322; doi:10.1002/cl2.70022)
Supplement: Supplementary file 1 — Supporting information. [file CL2-21-e70022-s001.docx]

# Appendices

**Appendix I - Example of Search Strategy**

PubMed

| #1 | TITLE/ABSTRACT (older* OR elder* OR geriatr* OR gerontol* OR senior* OR aging OR ageing OR “aged population”) |
| --- | --- |
| #2 | Aged[MeSH] OR "Aged, 80 and over"[MeSH] |
| #3 | TITLE/ABSTRACT (“digital health” OR “electronic health” OR “mobile health” OR “health technolog*” OR mhealth OR m-health OR ehealth OR e-health OR telehealth OR tele-health OR “digital technolog*” OR “mobile technolog*” OR “smart technolog*” OR “smart health technolog*” OR “computer-based technolog*” OR telemedicine OR tele-medicine OR tele-healthcare OR telecare OR tele-care OR telemonitoring OR tele-monitoring OR “medical informatics” OR “health informatics” OR “wireless technolog*” OR wearable* OR “assistive technolog*” OR gerontechnolog* OR telegeriatric* OR “health information technolog*” OR “health apps” OR “digital intervention*” OR “computer-based intervention*”) |
| #4 | "Digital Health"[MeSH Terms] OR Telemedicine[MeSH Terms] |
| #5 | TITLE/ABSTRACT (attitud* OR belief* OR norm* OR opinion* OR perception* OR perspective* OR view* OR knowledge* OR experience* OR reflect* OR educat* OR expertise OR value* OR barrier* OR difficult* OR limitation* OR restriction* OR drawback OR facilitat* OR enabler* OR aid OR factor* OR mindset OR willingness OR readiness OR acceptability OR acceptance OR usability OR preference* OR adoption) |
| #6 | Attitude[MeSH Terms] OR “Social Norms”[MeSH Terms] OR “Social Values”[MeSH Terms] |
| #7 | TITLE/ABSTRACT (review* OR “meta analys*” OR meta-analys* OR “meta synthes* OR meta-synthes* OR metasynthes* OR “evidence-based synthes*” OR “evidence-based analys*” OR “evidence synthes*”) |
| #8 | "systematic review"[MeSH Terms] |
| #9 | (#1 OR #2) AND (#3 OR #4) AND (#5 OR #6) AND (#7 OR #8) |
| Year | 2005 |

**Appendix II - Draft data extraction tools**

Extraction Tool 1 – Description of the included reviews

| **Review details** | |
| --- | --- |
| Citation details |  |
| Type of review |  |
| Objectives |  |
| Participants sociodemographic and health-related characteristics |  |
| Setting/context and geographical location |  |
| Description of concept under study |  |
| **Search strategy and primary studies details** | |
| Number and type of sources searched |  |
| Publication year range of included studies |  |
| Number of studies included |  |
| Type of study design of included studies |  |
| Country of origin of included studies |  |
| **Appraisal** | |
| Critical appraisal instrument used |  |
| Critical appraisal rating or interpretation |  |
| **Analysis** | |
| Method of analysis |  |
| **Comments** | |

Extraction Tool 2 – Description and classification of the attitudinal factors related to the use of digital technologies in health by older adults, described in reviews with qualitative components (related to research questions no. 1 and no. 2)

| **Review details** | |
| --- | --- |
| Citation details |  |
| **Findings relevant to the present overview of reviews** | |
| Participants sociodemographic and health-related characteristics |  |
| Setting/context and geographical location |  |
| Digital health technology of interest |  |
| Attitudinal factors reported by the review |  |
| Classification of each attitudinal factor into the categories of facilitators or barriers |  |
| **Comments** | |

Extraction Tool 3 - Description and classification of the attitudinal factors related to the use of digital technologies in health by older adults, described in reviews with quantitative components (related to research questions no. 1 and no. 2)

| **Review details** | |
| --- | --- |
| Citation details |  |
| **Findings relevant to the present overview of reviews** | |
| Participants sociodemographic and health-related characteristics |  |
| Setting/context and geographical location |  |
| Digital health technology of interest |  |
| Attitudinal factors reported by the review |  |
| Classification of each attitudinal factor into the categories of facilitators or barriers |  |
| **Comments** | |

Extraction Tool 4 - Attitudinal factors and technology use behaviour, and their relationship with the type of technology and the purpose and context of this technology use, described in reviews with qualitative components (related to research questions no. 3 and no. 4)

| **Review details** | |
| --- | --- |
| Citation details |  |
| **Findings relevant to the present overview of reviews** | |
| Participants sociodemographic and health-related characteristics |  |
| Setting/context and geographical location |  |
| Attitudinal factors reported by the review |  |
| Digital health technology of interest |  |
| Classification of digital health technology into one of the following categories:  - mobile technologies  - nonmobile technologies  - digital devices  - artificial intelligence technologies  - other (which?) |  |
| Context of the digital health technology use  - advancing health  - preventing illness  - providing treatment  - other (which?) |  |
| Purpose of the digital health technology use  - screening  - monitoring  - counselling  - remote interaction  - other (which?) |  |
| Significance/direction |  |
| **Comments** | |

Extraction Tool 5 - Attitudinal factors and technology use behaviour, and their relationship with the type of technology and the purpose and context of this technology use, described in reviews with quantitative components (related to research questions no. 3 and no. 4)

| **Review details** | |
| --- | --- |
| Citation details |  |
| **Findings relevant to the present overview of reviews** | |
| Participants sociodemographic and health-related characteristics |  |
| Setting/context and geographical location |  |
| Attitudinal factors reported by the review |  |
| Digital health technology of interest |  |
| Classification of digital health technology into one of the following categories:  - mobile technologies  - nonmobile technologies  - digital devices  - artificial intelligence technologies  - other (which?) |  |
| Context of the digital health technology use  - advancing health  - preventing illness  - providing treatment  - other (which?) |  |
| Purpose of the digital health technology use  - screening  - monitoring  - counselling  - remote interaction  - other (which?) |  |
| Heterogeneity of the included studies |  |
| If meta-analysis: effect size measure of the association between the reported attitudinal factors and certain types of digital health technology, with respective level of significance  If not meta-analysis: number and percentage of primary studies indicating a relationship between the attitudinal factor and certain type of digital health technology |  |
| Significance/direction |  |
| **Comments** | |
